# Contrasting Osmotic Stress Responses and Provenance Effects on Seed Germination and Seedling Performance in Two Andean Species

**DOI:** 10.1002/pei3.70151

**Published:** 2026-04-28

**Authors:** Claudia Patiño‐Uyaguari, Eduardo Chica, Selene Báez, Thomas Sibret, Ximena Palomeque

**Affiliations:** ^1^ Facultad de Ciencias Agropecuarias Universidad de Cuenca Cuenca Ecuador; ^2^ Departamento de Recursos Hídricos y Ciencias Ambientales Universidad de Cuenca Cuenca Ecuador; ^3^ Département de phytologie Faculté des sciences de l’agriculture et de l’alimentation, Université Laval Québec Canada; ^4^ Departamento de Biología, Facultad de Ciencias Escuela Politécnica Nacional Quito Ecuador; ^5^ Department of Environment Ghent University Ghent Belgium

**Keywords:** biomass allocation, drought, forest restoration, relative water content, stem water potential

## Abstract

The Andes are currently experiencing prolonged and frequent drought events presenting significant challenges to the natural ecosystems. This study examines how seed provenance and osmotic stress influence germination (radicle and cotyledon emergence) and seedling responses in *Oreocallis grandiflora* (tree) and *Salvia corrugata* (shrub), two species with significant potential for maintaining important ecological interactions in the forest. Seeds were collected from individuals in dry and wet provenances of high‐elevation mountain forest in Ecuador. Two independent experiments were conducted under controlled and greenhouse conditions. Results revealed marked differences in the drought response of both species. *O. grandiflora* exhibited greater sensitivity to osmotic stress during seed germination, and seedling mortality occurred within 10 days. However, few seeds from the dry provenance formed their cotyledonary leaves completely, while none from wet provenance germinated. In contrast, *S. corrugata* had successfully developed radicles and cotyledonary leaves under all osmotic stress treatments across both provenances. Seedlings of this species experienced osmotic stress but recovered within 60 days. Individuals exhibited early‐ stage adaptations to drought, including defoliation, stomatal conductance and transpiration regulation, and higher leaf relative water content, indicating greater drought tolerance. Provenance effects were strong in *O. grandiflora* but weak in *S. corrugata* . Our findings confirm species‐specific and provenance‐dependent responses to water limitation, emphasizing the importance of selecting appropriate species and seed sources to increase restoration success under climate change scenarios.

## Graphical abstract

*Oreocallis grandiflora* was highly drought‐sensitive, whereas *Salvia corrugata* recovered and showed adaptive traits. Provenance effects were strong in *O. grandiflora* but weak in *S. corrugata* . These results reveal species‐ and provenance‐dependent responses to water limitation, highlighting the importance of selecting suitable species and seed sources for restoration under climate change.

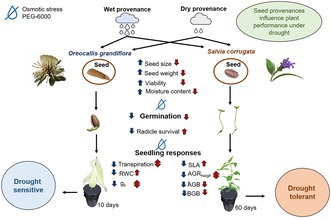

## 1 Introduction

Tropical Andean forests are biodiversity hotspots (Rahbek et al. 2019) that are increasingly threatened by climate change, which is expected to alter precipitation patterns and raise the frequency of droughts and floods (Motschmann et al. 2020). Drought can affect plant communities by altering species composition, plant performance, phenology, and ecological interactions (Gallagher and Campbell 2021; Jaworski et al. 2022; Thuma et al. 2023).

Several studies indicate that plant species employ different drought‐response strategies, including avoidance, tolerance, and escape (Kooyers 2015; Basu et al. 2016; Haghpanah et al. 2024). These strategies are mediated by plant functional traits and involve trade‐offs that shape performance under contrasting water conditions (Violle et al. 2007; Balachowski and Volaire 2018; Kühn et al. 2021). Species that lack effective expression of these strategies are often described as drought‐sensitive, exhibiting reduced performance and survival under water‐limited conditions (Laxa et al. 2019).

Additionally, provenance and the associated genetic diversity among populations may influence drought responses through variation in phenotypic plasticity and local adaptation (Oyanoghafo et al. 2023). Phenotypic plasticity allows plants to adjust their physiological and morphological traits in response to water availability (Zacharias et al. 2025), whereas local adaptation enables populations to achieve higher fitness in their home environments than in foreign ones (Tombesi et al. 2018; Gya et al. 2023). For instance, populations from historically dry provenances may exhibit greater performance under drought conditions (Barton et al. 2020), while populations from wetter environments may exhibit a deficiency in drought‐adaptive traits (Vasques et al. 2013), potentially influenced by epigenetic factors (Baskin and Baskin 2019). Furthermore, populations from regions with more variable precipitation often exhibit greater seed size variation, consistent with a bet‐hedging strategy that may enhance survival under unpredictable conditions (Gianella et al. 2021; Christie et al. 2022).

Drought responses are particularly critical during early life stages. At the seed level, plants of tropical montane forest have evolved drought adaptive traits that include dormancy, which prevents germination under unfavorable conditions (Athugala et al. 2021). Seed size may also contribute to drought tolerance; larger seeds provide stored resources to support the young seedlings' survival (Pérez‐Harguindeguy et al. 2013; Barczyk et al. 2024), while smaller seeds and orthodox might tolerate prolonged periods of water scarcity, as observed in tropical dry forest (Galindo‐Rodriguez and Roa‐Fuentes 2017).

At the seedling stage, several morpho‐physiological traits are widely used to assess plant responses to drought stress. Common morphological adaptations include reduced height, leaf size, and leaf area (Yang et al. 2021) and shifts in both above and below‐ground resource allocation (Chirino et al. 2017). Physiological adjustments involve stomatal regulation, restricting photosynthesis by limiting transpiration (Chastain et al. 2014; Zhang et al. 2022; Wu et al. 2022), reduced stem water potential (Golldack et al. 2011; Sánchez‐Piñero et al. 2023), and maintaining high levels of leaf relative water content (Soltys‐Kalina et al. 2016; Patanè et al. 2022). These traits allow seedlings to use water slowly and adjust physiological processes to tolerate drought (Devi and Reddy 2020), preventing death and supporting recovery after stress (Ruehr et al. 2019; Gebauer et al. 2023). Importantly, these responses can vary among provenances, as seedlings from wetter sites often show poor adjustment to drought due to limited exposure to water stress in their local environments (Vasques et al. 2013).

Despite the growing interest in understanding vegetation responses to climate change, knowledge of native species' responses to drought and the effects of seed provenance remains scarce in the Andes (Chirino et al. 2017; Cáceres et al. 2021). This knowledge gap limits our ability to predict species performance and design effective restoration strategies under increasing climatic stress. Therefore, this study explores the effects of water stress and seed provenance on seed traits, germination and seedling performance in two widely distributed woody Andean species*: Oreocallis grandiflora* and *Salvia corrugata*. We address the following hypothesis: (1) Seed germination (radicle and cotyledon emergence) decreases with increasing osmotic stress in both species; but seeds from the dry provenance show higher germination and radicle survival than those from the wet provenance, and (2) seedlings from dry provenance will show greater tolerance to drought, expressed in morphological and physiological responses. Our results provide insights into the mechanisms underlying early‐stage performance under water stress. These findings underscore the importance of considering seed provenance and functional traits when selecting species for restoration purposes in the context of climate change.

## 2 Materials and Methods

### 2.1 Species Selection

**FIGURE 1:**
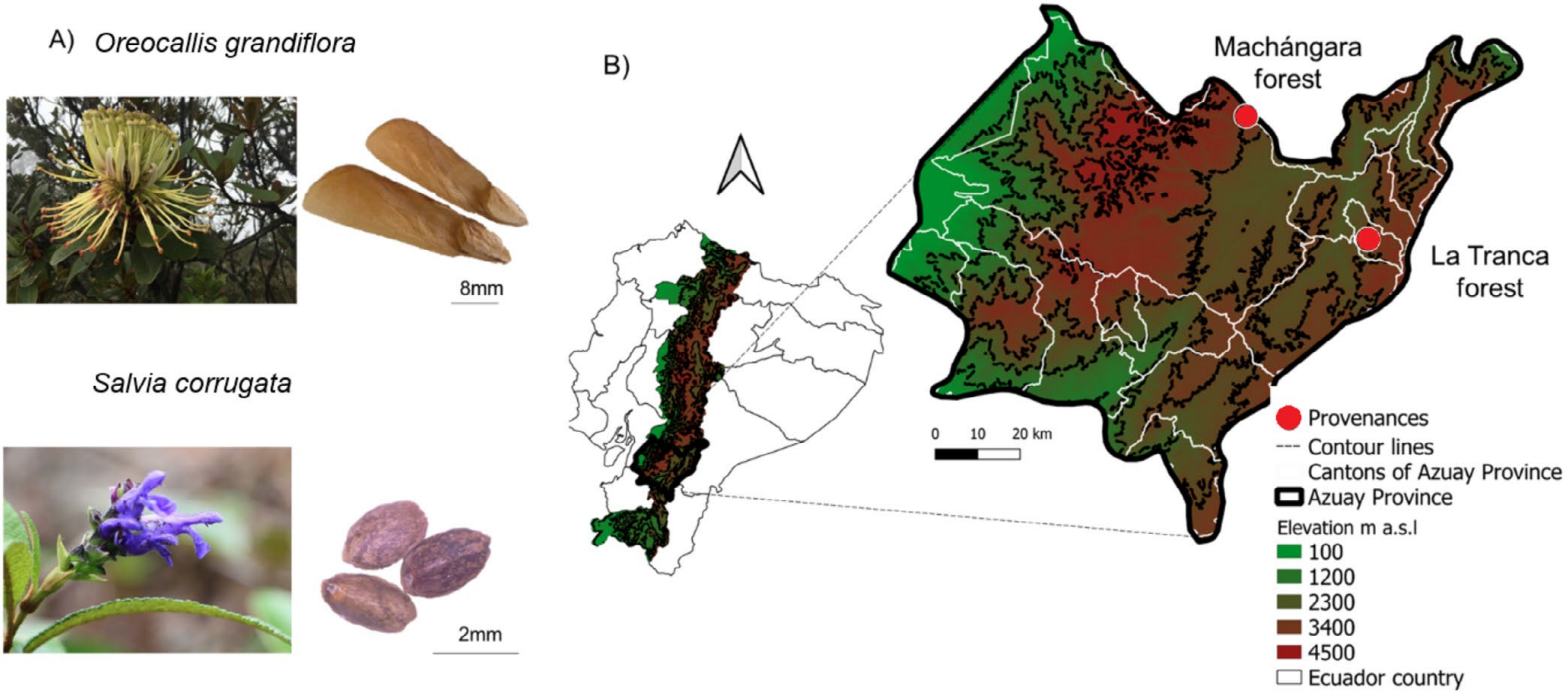
Species and provenances evaluated in this study. (A) Flowers and seeds of *O. grandiflora* (top) and *S. corrugata* (bottom). (B) Location of the provenances (red dots) from where seeds were collected: Wet provenance (Machángara) and dry provenance (La Tranca).

*Oreocallis grandiflora* (Lam.) R. Br. (Proteaceae, tree or shrub) and *Salvia corrugata* Vahl (Lamiaceae, shrub) are woody species native to Andean montane forests of Colombia, Ecuador, and Peru (Jørgensen and León‐Yánez 1999) (Figure 1A). Their combined range extends from 900 to 3600 m a.s.l. We selected these species due to their ecological importance in maintaining key interactions and their potential for use in restoration (Crespo et al. 2021).

### 2.2 Seed Collection

Seeds of both species were collected from two provenances differing in annual precipitation in high mountain forest of southern Ecuador (Azuay province). These are referred to as wet (Machángara forest, 3050–3180 m a.s.l.) and dry (La Tranca forest, 2840–3010 m a.s.l.) provenances (Figure 1B). Mean annual precipitation was estimated from WorldClim data (1970–2000) (Fick and Hijmans 2017), while field sensors provided temperature and humidity data for 2022–2023 (Table 1).

**TABLE 1:** Climatic description of two Andean forests located in the Azuay province, Ecuador.

Provenance	Altitude m a.s.l.	Annual precipitation (mm)	Annual temperature (°C)			Mean relative humidity (%)
			Mean	Min	Max	
Wet provenance	3050–3180	1143	10	3	20	97.70
Dry provenance	2840–3010	947	12	5	26	80.70

Between September and December 2022, ripe fruits were collected from at least 15 individuals per species at each location. Seeds were manually extracted, cleaned, and classified for seed description, germination test, and seedling growth experiments. Prior to experimentation, seeds were stored for 7 days at 5°C in hermetically sealed bags with silica gel.

### 2.3 Seed Characterization

Fresh seeds for each species and provenance were characterized following the International Seed Testing Association (ISTA 2024) guidelines, such as: seed weight (g), seed size (mm), moisture content (%), and initial viability (%). Weight was determined using four replicates of 100 seeds each. Seed size was assessed by measuring the length and width of 100 seeds using a stereo microscope (Nikon‐SMZ745T). Moisture content was determined using two replicates with 100 seeds each, which were weighed before and after exposure to 103°C for 17 h. Initial viability was tested on a separate seed lot using five replicates of 25 seeds in a 1% tetrazolium solution of 2.3.5 triphenyl tetrazolium chloride (TZ), classifying seeds as viable and non‐viable (unstained and empty seeds).

### 2.4 Osmotic Stress Induction at Seed and Seedlings Stages

Two independent experiments were conducted in the seed laboratory and greenhouse at the University of Cuenca, Ecuador. Osmotic stress was induced using polyethylene glycol (PEG‐6000, Sigma Aldridge, Sydney, NSW, Australia) to mimic conditions of reduced water availability during germination and initial seedling development. Three treatment levels of osmotic stress and a control were used to simulate scenarios of water availability in the medium where seeds and seedlings grow. The PEG solutions were prepared and calculated according to the equation proposed by Kaufmann and Michel (1973), where 0 MPa (control) represented a well‐irrigated plant, −0.2 MPa a mild to moderate water stress, −0.4 MPa a moderate water stress, and −0.6 MPa a slightly severe level of water stress (Haswell and Verslues 2015).

### 2.5 Seed Germination Experiment

A completely randomized design with four osmotic stress treatments (0, −0.2, −0.4 and −0.6 MPa) was conducted at room temperature (19°C ± 3°C). Seeds were placed on previously sterilized paper towels in Petri dishes and moistened with 5 mL of distilled water (0 MPa) or PEG‐6000 solutions, according to the treatments. To prevent evaporation, the Petri dishes were sealed with Parafilm. For each osmotic stress treatment and provenance, five replicates consisting of 25 seeds ( *O. grandiflora* ) or 50 seeds ( *S. corrugata* ) were used. Germination was monitored daily for 30 days and assessed in two phases: (1) radicle emergence and (2) cotyledonary leaves emergence. Radicle survival (%) was calculated as the proportion of seeds with alive radicles relative to the total number of seeds showing radicle emergence × 100. Radicle condition was assessed visually; radicles that were turgid and white, without signs of dehydration or necrosis, were classified as alive, whereas those that were brown, shriveled, or showed tissue decay were considered dead.

At the end of the experiment, a Tetrazolium test was performed on non‐germinated seeds to determine seed viability. Detailed information is available in the Supporting Informations (Figure S1; Table S1).

### 2.6 Seedling Growth Experiment

A completely randomized design was employed using four‐month‐old seedlings of both species produced under greenhouse conditions in peat substrate. A total of 96 seedlings per species were used, derived from seeds collected from dry and wet provenances. For *S. corrugata* , seeds were imbibed in 200 ppm GA_3_ for 48 h to accelerate germination and facilitate seedling production. Initial mean heights were 7.3 ± 1.9 cm for *O. grandiflora* and 9.4 ± 1.9 cm for *S. corrugata* . Seedlings were transplanted into 11 × 13 cm pots filled with a 1:1 sand–peat mix (Mahpara et al. 2022), 0.5 g of commercial fertilizer (10–12–18 + 2 Mg + 23S), and treated with PEG‐6000 osmotic solutions (0, −0.2, −0.4, −0.6 MPa). Osmotic solutions were thoroughly mixed with the substrate before potting, and pots were sealed with black plastic to reduce surface evaporation. Daily measurements of pot weight were taken over 10 days for *O. grandiflora* and 60 days for *S. corrugata* to determine cumulative transpiration.

#### 2.6.1 Physiological and Morphological Variables

Various physiological and morphological variables were measured 10 days after planting for *O. grandiflora* , and at 10, 45, and 60 days for *S. corrugata* . The shorter monitoring period for *O. grandiflora* was due to its earlier onset of severe dehydration compared to *S. corrugata* . Relative water content (RWC, %) was measured on one disc with a diameter of 0.5 mm from five randomly selected plants per treatment and provenance following Pieczynski et al. (2013). Discs were weighed fresh, after 24 h saturation in distilled water, and after drying at 60°C for 48 h. RWC was calculated according to Smart and Bingham (1974) protocol. Stem water potential (Ψ_stem_, MPa) was measured in three randomly selected plants per treatment using a pressure chamber between 12:00 and 15:00 (Argyrokastritis et al. 2015). Stomatal conductance (g_s_, mmol m^−2^ s^−1^) was recorded on well‐formed leaves from five plants per treatment on days 1, 5, 10, 15, 30, 45, and 60 of the experiment using a Porometer (SC‐1, Australia) between 12:00–14:00.

Specific leaf area (SLA, cm^2^ g^−1^) was measured on two well‐formed leaves from five plants per treatment and provenance. The absolute growth rate of height (AGR_height_) was estimated following Hunt (1990). Aboveground and belowground biomass (ABG and BGB, respectively) were determined from three plants per treatment by drying the biomass at 70°C for 48 h and then weighing it.

### 2.7 Data Analysis

For both experiments, two independent variables were considered: osmotic stress treatments (four‐levels) and provenances (two‐levels). For seeds traits, germination and radicle survival in both species and morpho‐physiological traits in seedlings for *O. grandiflora* , an analysis of variance (ANOVA) was used, followed by Tukey's HDS comparison test, where assumptions of normality and homogeneity of variance were achieved. For *S. corrugata* seedlings, we used a linear mixed model (LMM) using data collected at the end of the experiment. The osmotic stress treatments and provenances were considered as fixed effects, while each seedling was considered as random effect. The most appropriate model was selected based on the minimum values of Akaike Information Criterion (AIC) and Bayesian Information Criterion (BIC), compared with other models. The assumptions for the LMM such as random effects, normality, distributed experimental errors and homogeneous error variance were tested. All analyses were performed in R (R Core Team 2023) using the R package “lme4e” (Bates et al. 2015) and “lmerTest” (Kuznetsova et al. 2017).

## 3 Results

### 3.1 Seed Characteristics

Seeds *of O. grandiflora* from the wet provenance were significantly heavier, larger, and showed higher viability than those from dry provenance (*p* < 0.05). Seeds of *S. corrugata* from the dry provenance had higher weight and viability but were smaller than those from the wet provenance (*p* < 0.05), which also showed a higher proportion of non‐viable seeds. For both species, seed moisture content was higher in dry provenance, with significant differences (*p* < 0.05) observed only for *O. grandiflora* (Table 2).

**TABLE 2:** Seed characteristics of *O. grandiflora* and *S. corrugata* from wet and dry provenances in high mountain forest of the Andes in Southern Ecuador. Values represent means ± standard deviation.

Species	Seeds provenance	Weight (g)	Seed moisture content (%)	Seeds size		Viable seeds (%)	Non‐viable seeds (%)
				Length (mm)	Width (mm)		
*O. grandiflora*	Wet	3.4 ± 0.1^a^	25.9 ± 0.3^b^	8.8 ± 1.0^a^	6.0 ± 0.6^a^	89 ± 1.0^a^	11 ± 1.0^a^
	Dry	2.8 ± 0.1^b^	29.5 ± 0.6^a^	8.0 ± 0.9^b^	5.1 ± 0.7^b^	85 ± 1.5^b^	15 ± 1.5^a^
*S. corrugata*	Wet	0.10 ± 0.004^b^	15.6 ± 0.9^a^	2.4 ± 0.3^a^	1.3 ± 0.2^a^	26 ± 1.1^b^	74 ± 4.5^a^
	Dry	0.11 ± 0.003^a^	16.7 ± 0.3^a^	2.3 ± 0.3^b^	1.2 ± 0.2^b^	49 ± 2.2^a^	51 ± 8.6^b^

*Note:* Different letters indicate significance at *p* ≤ 0.05 according Tukey's HSD test.

### 3.2 Effect of Osmotic Stress on Seed Germination

**FIGURE 2:**
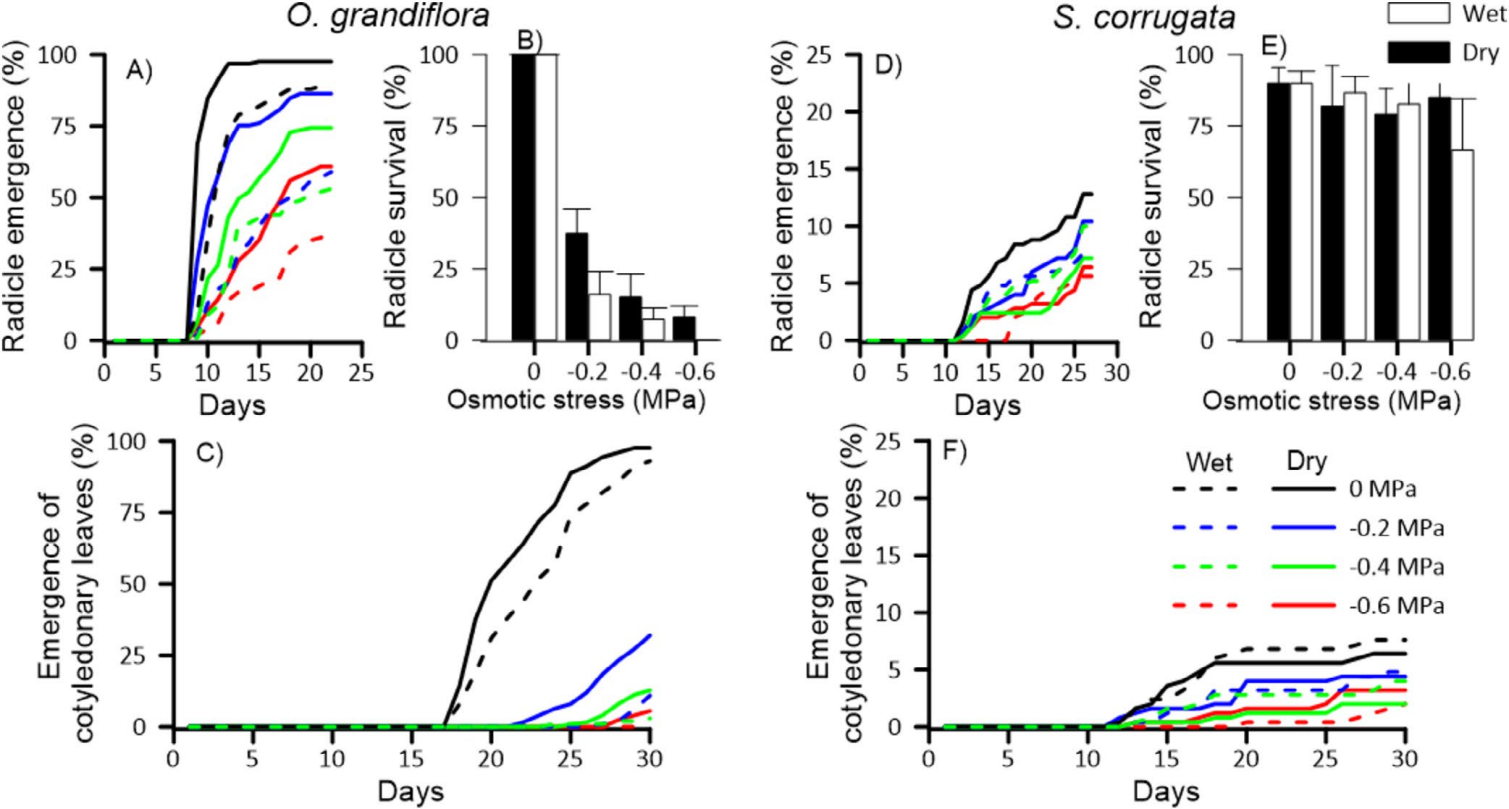
Cumulative radicle emergence and emergence of cotyledonary leaves (%) of *O. grandiflora* (A, C) and *S. corrugata* (D, F) seeds from wet (dashed lines) and dry (solid lines) provenances under different osmotic stress treatments. Bar graphs (B and E) show radicle survival (%) after 30 days for each provenance (white = wet; black = dry) ± SD; *n* = 5.

*O. grandiflora* seeds from both provenances reached more than 90% germination in the control treatment; however, seeds from the wet provenance required an additional 5 days to reach this percentage. Seeds germinated under osmotic stress had a reduced germination percentage compared to the control, and this reduction increased with the level of stress imposed (Figure 2A,C; Figure 5A). Radicle emergence under osmotic stress occurred in fewer days (< 10 days) and was consistently higher in seeds from the dry provenance (Figure 2A). Interestingly, we observed that in the osmotic stress treatments, some seeds with developed radicles died after 23 days, limiting cotyledon emergence to < 30% (Figure 2B,C). Germination and radicle survival were significantly affected by osmotic stress (*p* < 0.001), provenance (*p* < 0.05), and their interaction, particularly for radicle emergence (*p* < 0.001, Table 3).

In *S. corrugata* , the emergence of both radicle and cotyledonary leaves was low (< 15%) across all treatments and provenances (Figure 2D,F). Overall, germination was significantly affected by osmotic stress (*p* < 0.05; Table 3), however, it did not exhibit a clear trend across osmotic stress levels (Figure 5B). Some mortality of germinated seeds was observed even in the control treatment; however, radicle survival remained above 70% across all treatments (Figure 2E), with no statistically significant effects of treatment or provenance (*p* > 0.05, Table 3).

**TABLE 3:** Statistical analysis of the effect of osmotic stress and provenance on germination (radicle and cotyledonary leaf emergence) and radicle survival according to analysis of variance (ANOVA) for *O. grandiflora* and *S. corrugata* .

Species	Factors	Germination				Radicle survival (%)	
		Radicle emergence (%)		Emergence of cotyledonary leaves (%)			
		*F*	*p* value	*F*	*p* value	*F*	*p* value
*O. grandiflora*	Osmotic stress	3.15	**0.04**	16.39	**< 0.001**	12.16	**< 0.001**
	Provenance	0.95	0.34	9.11	**0.005**	5.89	**0.02**
	Osmotic stress: Provenance	16.65	**< 0.001**	1.22	0.32	1.2	0.33
*S. corrugata*	Osmotic stress	3.66	**0.02**	3.3	**0.03**	1.23	0.31
	Provenance	0.008	0.93	0.3	0.59	0.001	0.98
	Osmotic stress: Provenance	0.48	0.7	0.39	0.76	2.4	0.09

*Note:* The values highlighted in bold indicate statistical significance (*p* < 0.05).

### 3.3 Effect of Osmotic Stress on Physiological and Morphological Traits of Seedlings

**FIGURE 3:**
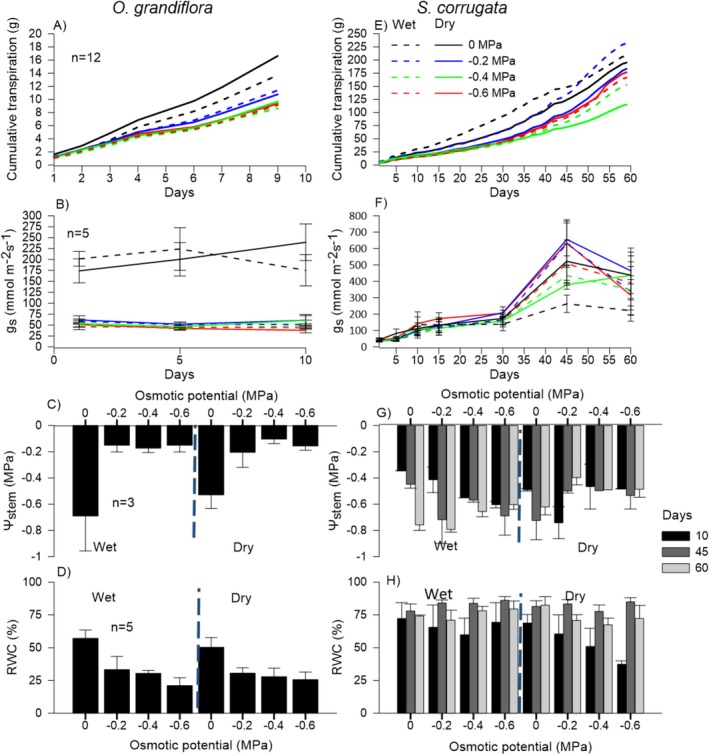
Physiological parameters in seedlings of *O. grandiflora* (A–D) and *S. corrugata* (E–H) under osmotic stress treatments in two provenances: Wet and dry. Daily cumulative transpiration (A, E); g_s_, Stomatal conductance (B, F); Ψ_stem_, Stem water potential (C, G); RWC, Relative water content (D, H). Different color bars represent each monitoring period; ± SD.

**FIGURE 4:**
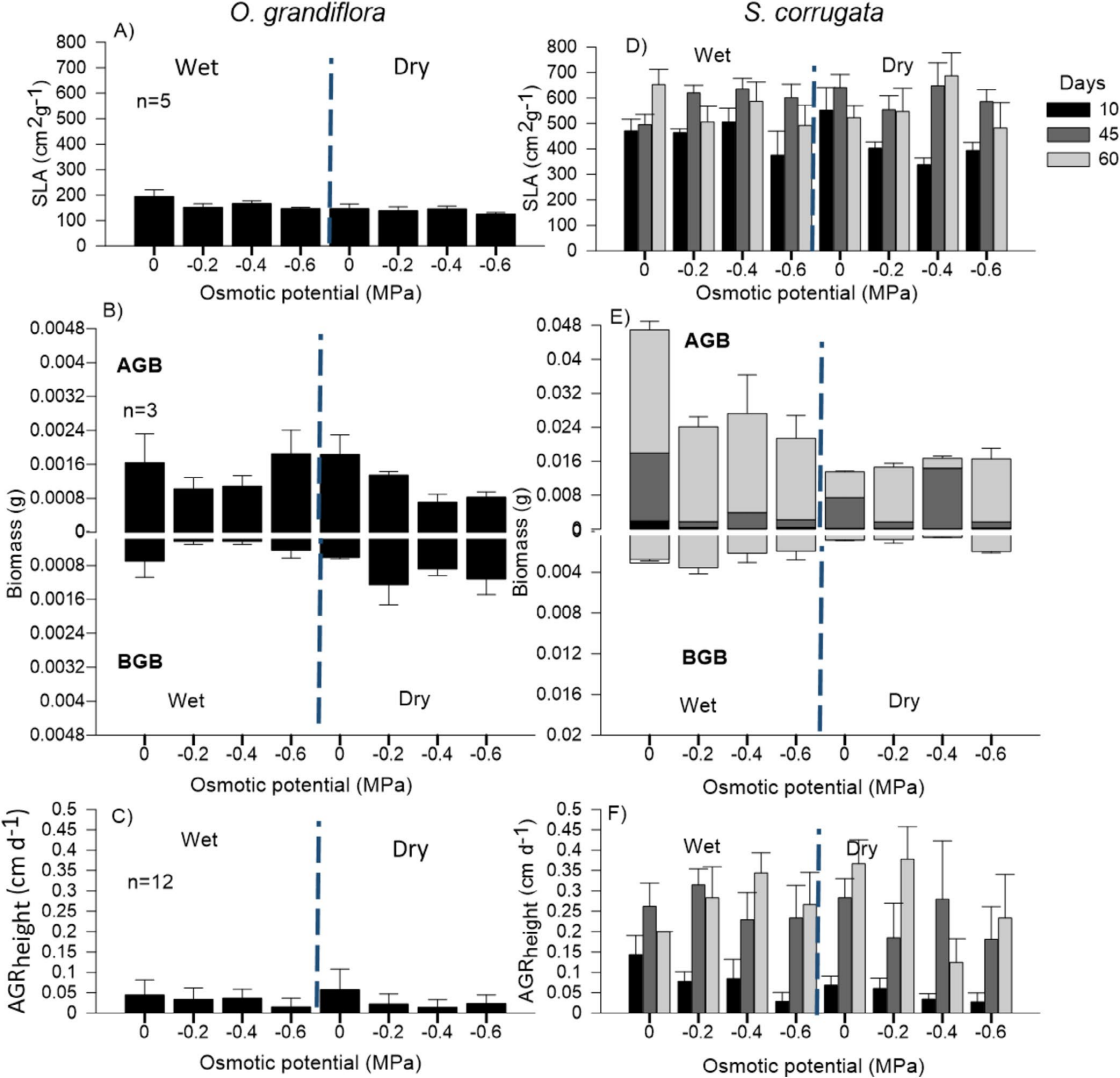
Morphological parameters in seedlings of *O. grandiflora* (A–C) and *S. corrugata* (D–F) under osmotic stress treatments in two provenances: Wet and dry provenances. SLA, Specific Leaf Area (A, D); AGB‐BGB, Above and belowground biomass (B, E); AGR_height_, Absolute growth rate of height (C, F). Different colored bars represent each monitoring period; ± SD.

Both species exhibited different morpho‐physiological responses to osmotic stress at the seedling stage. *O. grandiflora* seedlings from both provenances exhibited the highest cumulative transpiration, g_s_ and RWC under control treatment, together with Ψ_stem_ values near −0.7 MPa. However, seedlings subjected to osmotic stress treatments showed a clear decreasing trend in cumulative transpiration, g_s_, and RWC as osmotic stress increased. In contrast Ψ_stem_ remained relatively stable with higher values than −0.2 MPa (Figure 3A–D). These physiological variables differed significantly under osmotic stress (*p* < 0.001, Table 4), with no significant effect of provenance. SLA remained low across all treatments and provenances (< 200 cm^2^·g^−1^) (Figure 4A), with both factors showing a significant on this variable (*p* < 0.001, Table 5). In addition, seedlings from the dry provenance had significantly higher BGB than seedling from the wet provenance (*p* = 0.01, Figure 4B, Table 5). In contrast, AGR_height_ and AGB traits did not exhibit significant differences (*p* > 0.05, Table 5).

*S. corrugata* seedlings increased cumulative transpiration, g_s_, RWC, SLA, and AGR_height_ at 45 days across all treatments and provenances, followed by a decline at 60 days in most treatments, except for AGR_height_ (Figure 3E,F,H; Figure 4D,F). Seedlings from the dry provenance showed a smaller decline in Ψ_stem_ (−0.3 to −0.6 MPa) than those from the wet provenance (−0.5 to −0.8 MPa) (Figure 3G). At the end of the experiment, in both provenances the AGB exceeded BGB, however, the seedlings from the wet provenance allocated more AGB (Figure 4E). In general, provenance significantly affected especific morpho‐physiological variables (*p* < 0.05; Tables 4 and 5), whereas other variables were unaffected by either factor (*p* > 0.05; Tables 4 and 5). Interestingly, this species exhibited a recovery phase in most of the morphological and physiological variables, which was more evident at 45 days (Figures 3E,F,H and 4D,F; Figure S2).

**TABLE 4:** Statistical analysis of the effect of osmotic stress (MPa) and seed provenance on physiological parameters (Cumulative transpiration; g_s_, Stomatal conductance; Ψ_stem_, Stem water potential; RWC, Relative water content) according to analysis of variance (ANOVA) for seedlings of *O. grandiflora* and Linear Mixed Model (LMM) for seedlings of *S. corrugata* .

Species	Factors	Cumulative transpiration (g)		g_s_ (mmol m^−2^ s^−1^)		Ψ_stem_ (MPa)		RWC (%)	
		*F*	*p* value	*F*	*p* value	*F*	*p* value	*F*	*p* value
*O. grandiflora*	Osmotic stress	27.44	**< 0.001**	225.17	**< 0.001**	23.40	**< 0.001**	15.45	**< 0.001**
	Provenance	3.72	0.054	0.40	0.53	0.80	0.39	0.30	0.59
	Osmotic stress: Provenance	1.85	0.14	0.12	0.95	1.01	0.42	0.49	0.69
*S. corrugata*	Osmotic stress	1.4739	0.30	0.26	0.85	1.47	0.30	0.67	0.58
	Provenance	9.1163	**0.02**	1.45	0.24	9.12	**0.02**	0.31	0.59
	Osmotic stress: Provenance	0.5514	0.66	0.76	0.53	0.55	0.66	0.92	0.45

*Note:* The values highlighted in bold indicate statistical significance (*p* < 0.05).

**TABLE 5:** Statistical analysis of the effect of osmotic stress and seed provenance on morphological parameters (SLA, Specific leaf area; AGB, aboveground biomass; BGB, belowground biomass; AGR_height_, Absolute growth rate) according to Analysis of Variance (ANOVA) for seedlings of *O. grandiflora* and Linear Mixed Model (LMM) for seedlings of *S. corrugata* .

Species	Factors	SLA (cm^2^g^−1^)		AGB (g)		BGB (g)		AGR_height_ (cm d^−1^)	
		F	*p* value	F	*p* value	F	*p* value	F	*p* value
*O. grandiflora*	Osmotic stress	7.86	**< 0.001**	1.79	0.19	0.31	0.82	1.82	0.15
	Provenance	22.62	**< 0.001**	0.71	0.41	9.86	**0.01**	0.07	0.79
	Osmotic stress: Provenance	1.89	0.14	1.41	0.28	1.70	0.21	0.64	0.59
*S. corrugata*	Osmotic stress	5.55	**0.001**	0.48	0.71	2.26	0.21	0.94	0.43
	Provenance	0.001	0.96	18.75	**0.002**	26.84	**0.03**	0.06	0.81
	Osmotic stress: Provenance	3.02	**0.04**	1.84	0.22	4.14	0.20	0.94	0.43

*Note:* The values highlighted in bold indicate statistical significance (*p* < 0.05).

## 4 Discussion

We found that the two species responded differently to osmotic stress during radicle and cotyledon emergence, and seedling development. Provenance effects were strong in *O. grandiflora* but weak in *S. corrugata* , with populations from dry provenances being less affected by drought. Our findings highlight the importance of selecting appropriate species and seed provenances when planning restoration efforts to enhance plant establishment under drought conditions.

### 4.1 Seed Characteristics and Effects of Osmotic Stress on Germination

Radicle emergence decreased with increasing osmotic stress in both species. However, *S. corrugata* showed higher radicle survival and faster cotyledon emergence than *O. grandiflora* . Earlier cotyledon deployment might be related to access to stored reserves, support metabolic activity, and contribute to osmotic regulation during seedling establishment (Batool et al. 2022; Zhao et al. 2024; Li et al. 2026). In contrast, *O. grandiflora* exhibited low germination and impaired cotyledon development, which might be attributed to a limited internal capacity for osmotic adjustment (Zhang et al. 2022). PEG induced low hydration and may disrupt enzymatic activities and deplete reserves essential for radicle emergence (Saux et al. 2020). Similar results were reported in *Polylepis australis* and *Escallonia cordobensis* under combined heat and osmotic stress (Cáceres et al. 2021).

Osmotic stress had a strong effect on seeds of *O. grandiflora* from the wet provenance. These seeds were larger and had higher initial viability than those from the dry provenance, contradicting the theory that larger seeds perform better under drought conditions (Pérez‐Harguindeguy et al. 2013). This may reflect local adaptation of the mother plant to specific environmental conditions (Vivas et al. 2020; Vancostenoble et al. 2022). Conversely, seeds from the dry provenance with better germination are more likely to establish under limited water availability (Pennington et al. 2021; Magni et al. 2023). In general, this species is considered desiccation‐tolerant based on its seed moisture content, maintaining high germination capacity and viability over one year (Palomeque et al. 2020). However, our results reveal high sensitivity to osmotic stress.

In contrast, *S. corrugata*, despite low initial viability and germination, produced complete seedlings under osmotic stress conditions regardless of provenance, indicating phenotypic plasticity. Seed traits such as small seed size, low seed moisture content, seed dormancy which was observed in this study, and the presence of pectinaceous mucilage in the seed coat are consistent with a drought‐tolerant strategy. This trait has also been reported in other species of the genus *Salvia* (Punia and Dhull 2019; Mutlu et al. 2023). Hence, mucilage may reduce dehydration, preserve viability, and aid germination mainly in habitats with low soil moisture (Nazari 2021; Sohrabizadeh et al. 2023; Geneve et al. 2017).

### 4.2 Effects of Osmotic Stress on Physiological and Morphological Traits in Seedlings Performance

Overall, seedlings of the two species exhibited different drought‐response strategies associated with provenance (Figure 6). Provenance influenced osmotic stress responses, with stronger effects in *O. grandiflora* than in *S. corrugata* . Although *O. grandiflora* was more sensitive to stress (Figure 6A), populations from dry provenances outperformed those from wet provenances, suggesting local adaptation (Barton et al. 2020). In contrast, *S. corrugata* showed weaker provenance effects and greater environmental responsiveness, likely associated with phenotypic plasticity (Zacharias et al. 2025) (Figure 6B). The response mechanisms of each species are discussed below.

**FIGURE 5:**
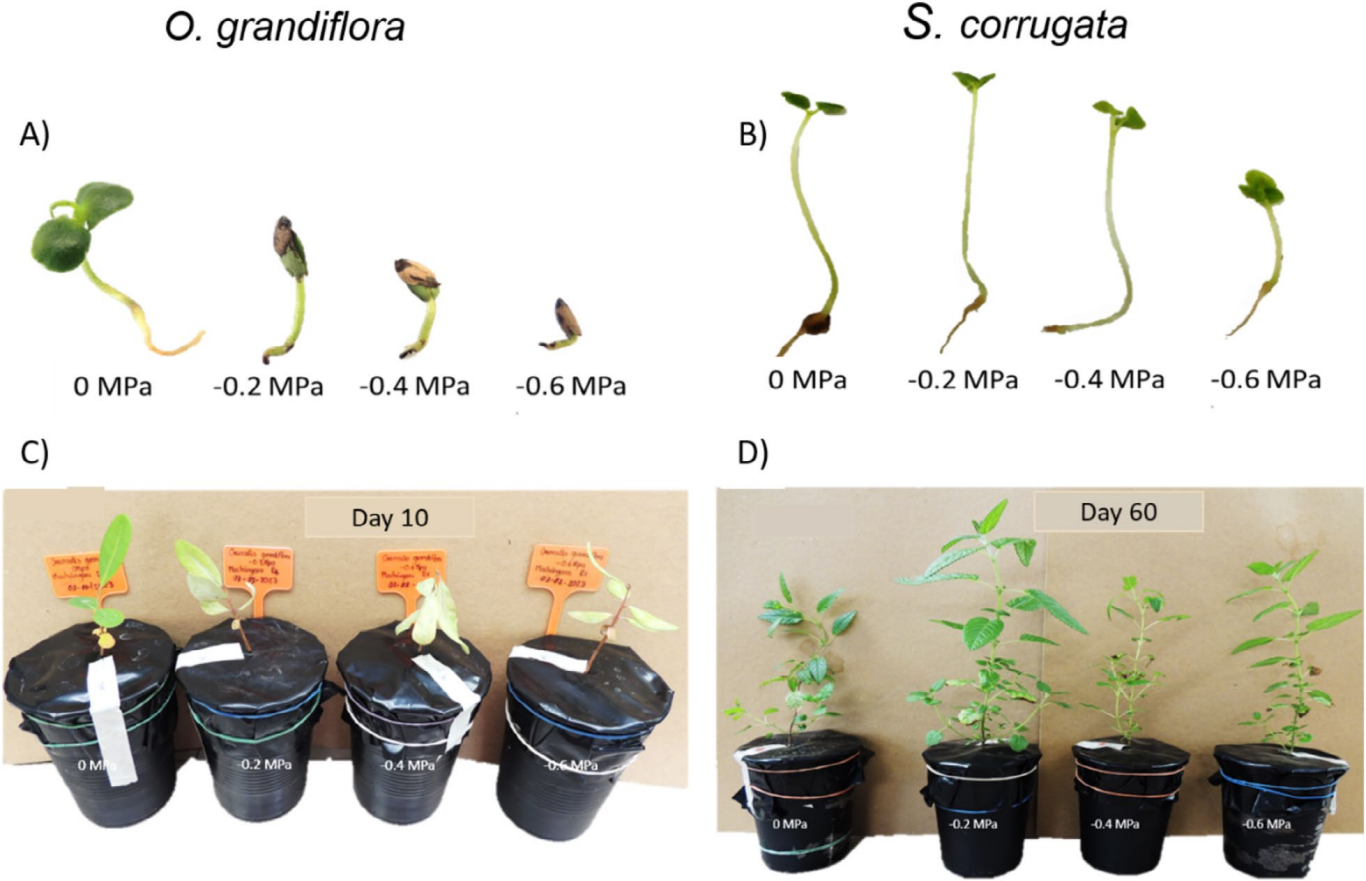
Representative images of seed germination (A, B) and seedling growth (C, D) under osmotic stress treatments, independent of provenance, in *O. grandiflora* (A, C) and *S. corrugata* (B, D). Seeds and seedlings are shown across increasing osmotic stress levels (0, −0.2, −0.4, and −0.6 MPa). Panels A and B illustrate seeds at 30 days after germination, whereas panels C and D show seedling development at 10 and 60 days, respectively.

*O. grandiflora* seedlings showed marked reductions in g_s_, RWC, and transpiration in response to increasing osmotic stress, regardless of provenance, suggesting a response common to many drought‐sensitive species. Although some morphological traits (e.g., SLA and BGB) differed among provenances, these differences did not enhance survival, as seedlings died within 10 days (Figure 5C), showing clear signs of dehydration as evidenced by low RWC. The RWC reduction may indicate poor compensation of water lost through plant transpiration (Zahedyan et al. 2022; Asadi and Eshghizadeh 2021), leading to hydraulic failure and premature mortality (Mantova et al. 2022; McDowell et al. 2022; Bouyer et al. 2023; Gea‐Izquierdo et al. 2023). Our results indicated higher Ψ_stem_ but lower RWC, suggesting a response pattern distinct from other studies. This could be an indicator of early xylem embolism in *O. grandiflora* seedlings caused by air bubble formation (Hölttä et al. 2009), accompanied by a transient capacitive effect of cavitation (Hölttä et al. 2012).

Conversely, seedlings of *S. corrugata* exhibited drought‐tolerant responses, characterized by higher RWC and stomatal conductance regulation, indicating a greater capacity to cope with water stress, likely supported by higher phenotypic plasticity (Férriz et al. 2023). This tolerance was further evidenced by survival across all treatments for over 60 days (Figure 5D). Similar results were reported in *S. virgata* (Khosrojerdi et al. 2023) and *S. officinalis* (Savi et al. 2013).

The tolerance of *S. corrugata* to water stress could be due to its osmotic adjustment ability (Saha et al. 2022), whereby plants accumulate solutes or osmolytes (osmoprotectants), aiding stress perception and transmitting signals as a defense mechanism (Ozturk et al. 2021; Khan et al. 2023). These internal mechanisms have been well described in *S. fruticosa* (Varela‐Stasinopoulou et al. 2023), *S. virgata* (Khosrojerdi et al. 2023), *S. abrotanoides, S. yangii* (Khodadadi et al. 2022) and *S. nemorosa* (Bayat and Moghadam 2019). Additionally, abscisic acid (ABA) and other phytohormones such as brassinosteroid, cytokinin, strigolactone also regulate water stress responses (Felemban et al. 2019; Iqbal et al. 2022; Bawa et al. 2023; Wang et al. 2023). Together, these mechanisms suggest an integrated physiological strategy that enables *S. corrugata* to withstand drought conditions during early developmental stages.

Interestingly, *S. corrugata* showed early stress signs, such as leaf senescence and defoliation (Figure S2) which are considered part of an adaptive strategy to reduce water loss (Aloryi et al. 2023). Unexpectedly, seedlings recovered after 45 days; similar findings were reported by Abate et al. (2021) in *S. ceratophylloides* and *S. officinalis* , which recovered hydraulic conductance. Plant recovery may also be mediated by non‐structural carbohydrates (NSCs), which provide energy and carbon to sustain metabolism, repair hydraulic systems, and support osmoregulation (Tomasella et al. 2019; Ozturk et al. 2021; Rostampour et al. 2023), thereby preventing seedling mortality.

**FIGURE 6:**
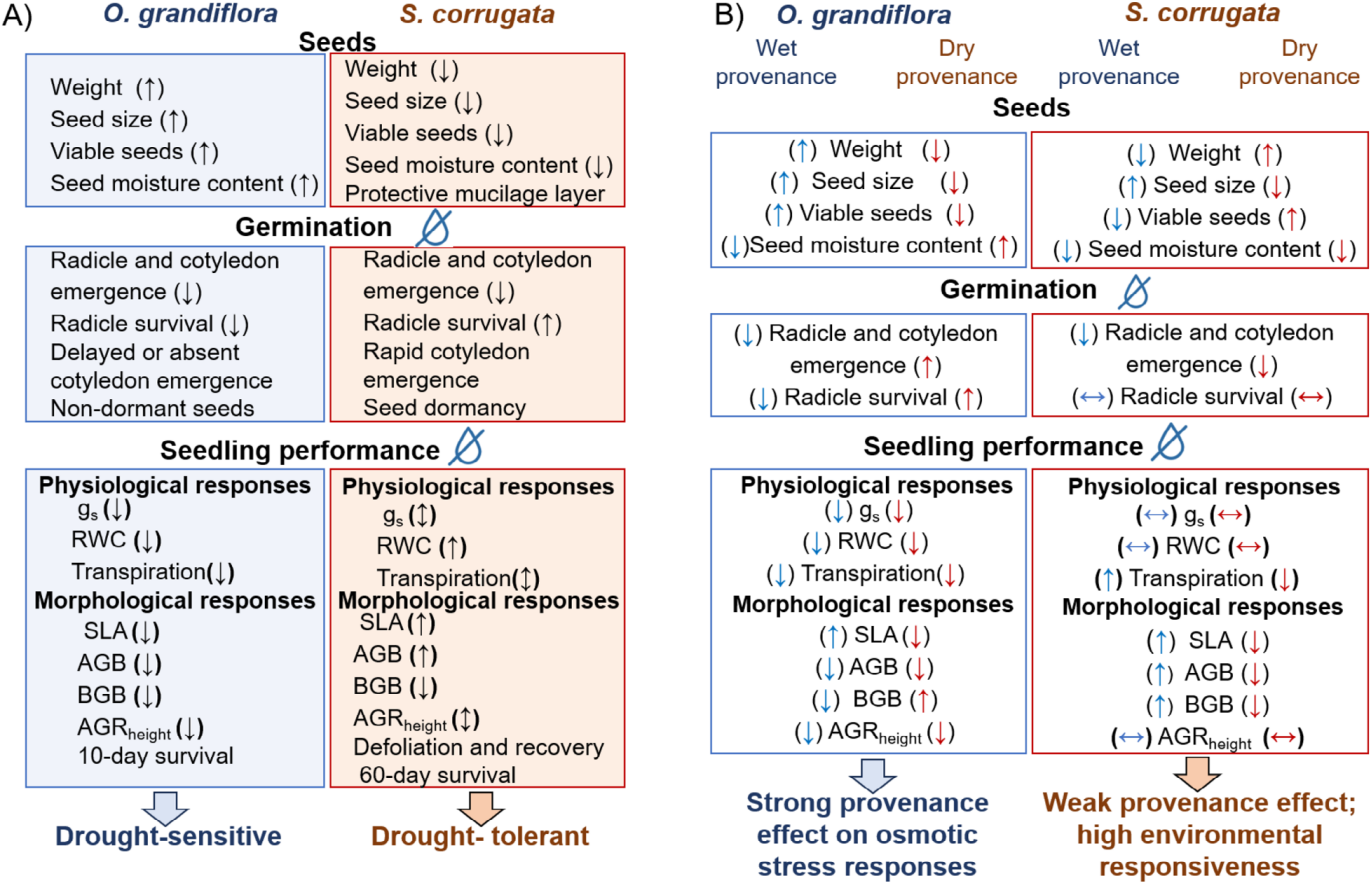
Summary of seed traits, germination, and seedling responses to osmotic stress and provenance in *Oreocallis grandiflora* and *Salvia corrugata*. (A) Species‐specific differences across early developmental stages, indicating greater drought sensitivity in *O. grandiflora* and higher drought tolerance in *S. corrugata* . (B) Effects of provenance (wet = blue arrows; dry = red arrows) on trait responses. *O. grandiflora* showed stronger provenance‐dependent responses, with dry‐provenance populations performing better under stress, whereas *S. corrugata* showed weaker provenance effects and high environmental responsiveness. Arrows indicate the direction of trait variation under osmotic stress across provenances (↑ increase, ↓ decrease, ↔ no change, ↕ variable response).

This study highlights the role of provenance and the integration of functional traits in understanding plant responses to drought as well as predicting species performance and survival. Our findings have significant implications for the success of ecological restoration programs in the Andean region. Based on the evidence presented here, *O. grandiflora* is expected to show poor seedling establishment under drought conditions due to its high sensitivity during early developmental stages at both the seed and seedling stages (Figure 6A). However, it is crucial to maintain seed sources for plant production in active restoration efforts as close to the restoration site as possible to promote better growth and survival rates in the local environment.

*S. corrugata* is a promising candidate for restoration due to traits that enhance survival during early developmental stages under drought. These traits include small seed size, low seed moisture content, radicle survival, rapid cotyledon emergence, osmotic adjustment, high RWC, and efficient stomatal regulation (Figure 6A). Such characteristics may support successful establishment in the changing environmental conditions of the Andes. However, further field studies are essential to confirm these results over the long term. We recommend extending our experiments to other species to broaden the pool of candidates for drought‐prone restoration and improve restoration success under future climate scenarios.

## Funding

This work was funded by the Swiss National Science Foundation (IZSTZ0_199379/1) and by the Vice‐Rectorate of Research at the University of Cuenca (Project 252 and 346) through the project *Experimental Network Ecology and Restoration*, Exper‐net.

## Conflicts of Interest

The authors declare no conflicts of interest.

## Supporting information


**Figure S1:** Effects of osmotic stress treatments and seeds provenance (wet and dry provenances) on viable (%) and non‐viable (%) seeds post germination of *O. grandiflora* (A) and *S. corrugata* (B), *n* = 5.
**Figure S2:** Recovery process of *Salvia corrugata* under osmotic stress treatments in two provenances: wet and dry at 5, 10, 25, 35, 45, and 60 days of the experiment.
**Table S1:** Statistical analysis of the effect of osmotic stress and provenance on viable and non‐viable seeds post germination according to Analysis of Variance (ANOVA) for *O. grandiflora* and *S. corrugata* .

## Data Availability

The data that support the findings of this study are openly available in Zenodo at https://doi.org/10.5281/zenodo.17945031.
