# Supplementary material for: Contrasting Osmotic Stress Responses and Provenance Effects on Seed Germination and Seedling Performance in Two Andean Species
Source: Plant Environ Interact. 2026 Apr 28;7(3):e70151. doi: 10.1002/pei3.70151 (PMC13122259; doi:10.1002/pei3.70151)
Supplement: Supplementary file 1 — Figure S1: Effects of osmotic stress treatments and seeds provenance (wet and dry provenances) on viable (%) and non‐viable (%) seeds post germination of O. grandiflora (A) and S. corrugata (B), n = 5. Figure S2: Recovery process of Salvia corrugata under osmotic stress treatments in two provenances: wet and dry at 5, 10, 25, 35, 45, and 60 days of the experiment. Table S1: Statistical analysis of the effect of osmotic stress and provenance on viable and non‐viable seeds post germination according to Analysis of Variance (ANOVA) for O. grandiflora and S. corrugata . [file PEI3-7-e70151-s001.docx]

**Osmotic stress tolerance relates to provenance in two key species for hummingbirds in the Andes**

Claudia Patiño-Uyaguari^ab^*, Eduardo Chica^a,^ Selene Báez^c^, Thomas Sibret^d^, Ximena Palomeque^a,b^

1. Effect of osmotic stress on seed viability

MATERIALS AND METHODS

At the end of the experiment of seed germination under stress treatments, a Tetrazolium test was performed on non-germinated seeds. Two categories were defined: 1) viable seeds and 2) non-viable seeds, which included unstained seeds, seeds without embryos and rotten seeds showing signs of fungal infection.

**Data Analysis**

For viable and non- viable seeds in both species an analysis of variance (ANOVA) was used, followed by Tukey’s HDS comparison test, where assumptions of normality and homogeneity of variance were achieved.

**RESULTS**

Compared with the initial tetrazolium test (Table 2), *O. grandiflora* seeds from the wet provenance were most affected by osmotic stress, with >30% non-viable seeds (Supplementary Figure1). Seed viability was significantly influenced by treatment and provenance (p < 0.001; Supplementary Table 1). In *S. corrugata*, all factors affected viability, with provenance showing the strongest effect (p < 0.001; Supplementary Table 1).


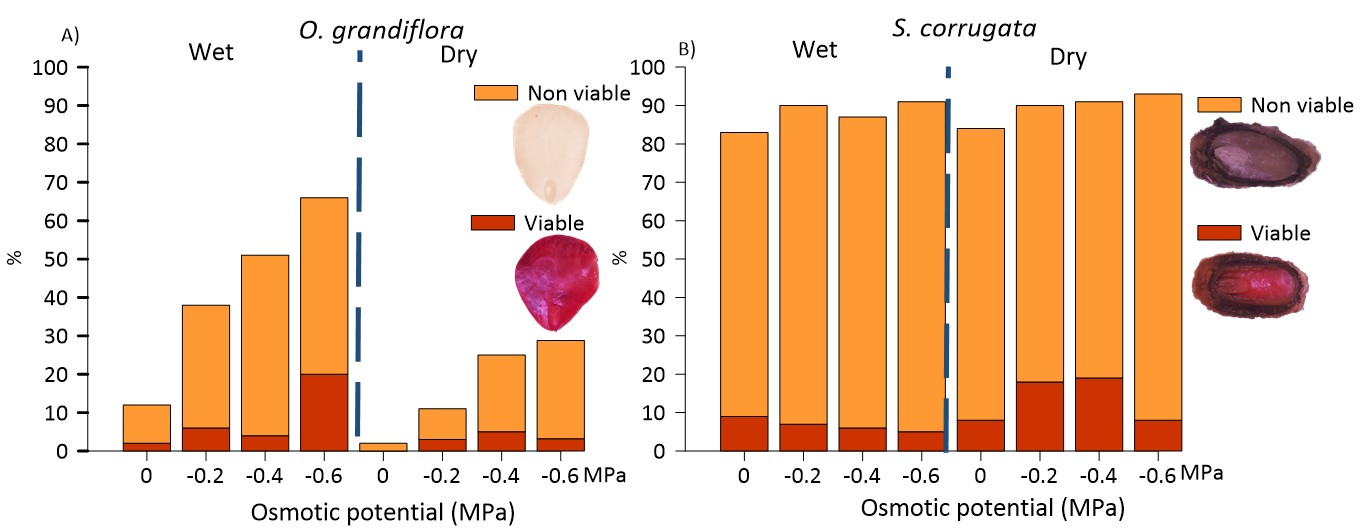


**Supplementary Figure 1.** Effects of osmotic stress treatments and seeds provenance (wet and dry provenances) on viable (%) and non-viable (%) seeds post germination of *O. grandiflora* (A) and *S. corrugata* (B), n=5.

**Supplementary Table 1.** Statistical analysis of the effect of osmotic stress and provenance on viable and non-viable seeds post germination according to Analysis of Variance (ANOVA) for *O. grandiflora* and *S. corrugata*.

| **Species** | **Factors** | **Viable seeds (%)** | | **Non-viable seeds (%)** | |
| --- | --- | --- | --- | --- | --- |
|  |  | ***F*** | ***p value*** | ***F*** | ***p value*** |
| ***O. grandiflora*** | **Osmotic stress** | 1.42 | 0.26 | 12.65 | **< 0.001** |
|  | **Provenance** | 1.73 | 0.20 | 39.83 | **< 0.001** |
|  | **Osmotic stress: Provenance** | 1.10 | 0.36 | 1.89 | 0.15 |
| ***S. corrugata*** | **Osmotic stress** | 3.33 | **0.03** | 3.09 | **0.04** |
|  | **Provenance** | 14.21 | **< 0.001** | 4.03 | 0.05 |
|  | **Osmotic stress (MPa): Provenance** | 4.04 | **0.02** | 0.71 | 0.55 |

1.
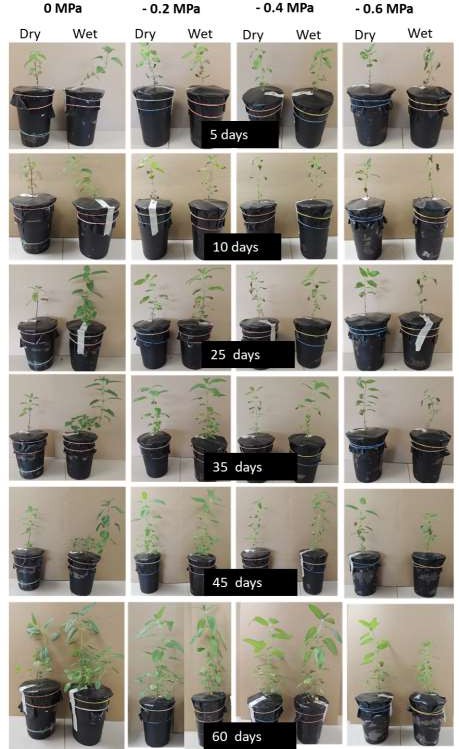
**Recovery process of *Salvia corrugata* under osmotic stress treatments in two provenances**

**Supplementary Figure 2.** Recovery process of *Salvia corrugata* under osmotic stress treatments in two provenances: wet and dry at 5, 10, 25, 35, 45, and 60 days of the experiment.
